# Flow-controlled ventilation maintains gas exchange and lung aeration in a pediatric model of healthy and injured lungs: A randomized cross-over experimental study

**DOI:** 10.3389/fped.2022.1005135

**Published:** 2022-09-09

**Authors:** Álmos Schranc, Ádám L. Balogh, John Diaper, Roberta Südy, Ferenc Peták, Walid Habre, Gergely Albu

**Affiliations:** ^1^Unit for Anesthesiological Investigations, Department of Anesthesiology, Pharmacology, Intensive Care and Emergency Medicine, University of Geneva, Geneva, Switzerland; ^2^Department of Medical Physics and Informatics, University of Szeged, Szeged, Hungary; ^3^Pediatric Anesthesia Unit, Department of Anesthesiology, Pharmacology, Intensive Care and Emergency Medicine, University Hospitals of Geneva, Geneva, Switzerland; ^4^Division of Anesthesiology, Department of Anesthesiology, Pharmacology, Intensive Care and Emergency Medicine, University Hospitals of Geneva, Geneva, Switzerland

**Keywords:** flow-controlled ventilation, pediatric model, respiratory mechanics, gas exchange, lung aeration, respiratory distress syndrome

## Abstract

Flow-controlled ventilation (FCV) is characterized by a constant flow to generate active inspiration and expiration. While the benefit of FCV on gas exchange has been demonstrated in preclinical and clinical studies with adults, the value of this modality for a pediatric population remains unknown. Thus, we aimed at observing the effects of FCV as compared to pressure-regulated volume control (PRVC) ventilation on lung mechanics, gas exchange and lung aeration before and after surfactant depletion in a pediatric model. Ten anesthetized piglets (10.4 ± 0.2 kg) were randomly assigned to start 1-h ventilation with FCV or PRVC before switching the ventilation modes for another hour. This sequence was repeated after inducing lung injury by bronchoalveolar lavage and injurious ventilation. The primary outcome was respiratory tissue elastance. Secondary outcomes included oxygenation index (PaO_2_/FiO_2_), PaCO_2_, intrapulmonary shunt (Qs/Qt), airway resistance, respiratory tissue damping, end-expiratory lung volume, lung clearance index and lung aeration by chest electrical impedance tomography. Measurements were performed at the end of each protocol stage. Ventilation modality had no effect on any respiratory mechanical parameter. Adequate gas exchange was provided by FCV, similar to PRVC, with sufficient CO_2_ elimination both in healthy and surfactant-depleted lungs (39.46 ± 7.2 mmHg and 46.2 ± 11.4 mmHg for FCV; 36.0 ± 4.1 and 39.5 ± 4.9 mmHg, for PRVC, respectively). Somewhat lower PaO_2_/FiO_2_ and higher Qs/Qt were observed in healthy and surfactant depleted lungs during FCV compared to PRVC (*p* < 0.05, for all). Compared to PRVC, lung aeration was significantly elevated, particularly in the ventral dependent zones during FCV (*p* < 0.05), but this difference was not evidenced in injured lungs. Somewhat lower oxygenation and higher shunt ratio was observed during FCV, nevertheless lung aeration improved and adequate gas exchange was ensured. Therefore, in the absence of major differences in respiratory mechanics and lung volumes, FCV may be considered as an alternative in ventilation therapy of pediatric patients with healthy and injured lungs.

## Introduction

Maintaining adequate lung aeration and gas exchange is paramount during mechanical ventilation in the perioperative period. Lung aeration is particular important in children as compared to adults, as children are more susceptible to airway closure (1) and subsequent deterioration (2) in respiratory function. Furthermore, increased chest-wall compliance and enhanced elastic recoil of lung parenchyma make children more prone to developing atelectasis and ventilation heterogeneity than adults (3). Various modalities have been recently suggested to tackle this challenge without increasing the risk of excessive lung tissue stress and strain (4–6) in both healthy and injured lungs.

A novel ventilation modality, flow-controlled ventilation (FCV), is based on the application of a constant, low flow that generates a slow prolonged inspiration and a controlled linear expiration. The constant inspiratory and expiratory flow ensures a linear rise and fall in airway pressures with an inspiratory-to-expiratory time ratio (I:E) of 1:1. Furthermore, during the active expiration, the respirator decelerates the central airflow. This ventilation modality has been shown to reduce energy dissipation in the respiratory system (7, 8), and subsequently improve ventilation distribution both experimentally and clinically (9–12). Since FCV can be initiated through an ultra-thin (outer diameter: 4.4 mm, inner diameter: 2.4 mm) endotracheal tube that requires minimum space and provides controlled ventilation as opposed to jet ventilation, the particular advantage of this modality was primarily demonstrated in ear, nose and throat surgeries (13). While these surgical interventions are frequently used for children, previous studies assessing the efficiency of FCV have been limited to adults; therefore, there is an essential lack of knowledge on the potential pediatric application of this ventilation modality.

We aimed at investigating the feasibility of FCV in a pediatric animal model while using a standard endotracheal tube that allows the assessment of respiratory mechanical changes between the different ventilation modalities. We also aimed at comparing the gas exchange and lung ventilation profile of FCV to a ventilation modality conventionally applied in pediatric anesthesia: the pressure-regulated volume control (PRVC) mode. To characterize the applicability of FCV to injured lungs, experiments were performed in healthy and surfactant-depleted lungs. We hypothesized that FCV can be adapted to a pediatric setting to prevent airway closure, the development of atelectasis whilst maintaining lung aeration comparable to PRVC in both healthy and injured lungs.

## Materials and methods

### Ethical considerations

The experimental protocol was approved by the Animal Welfare Committee of the Canton of Geneva (Rue Adrien-Lachenal 8, 1207 Genève) and the Experimental Committee of the University of Geneva, Rue du Général Dufour 24, 1211 Geneva, Switzerland (no. GE 66/20; Prof. Doron Merkler, Academic Director of Animal Experimentation), on 12 May 2020. All procedures were performed in accordance with current Swiss animal protection laws (LPA, RS455). The current report follows the Animal Research Reporting of *in vivo* Experiments (ARRIVE) guidelines (14). The experiments were performed between 31 August and 11 September 2020.

### Animals and preparations

Four-week-old piglets (male *n* = 5, female *n* = 5; body weight: 10.4 ± 0.2 kg) were involved in our study. The animals were premedicated by the intramuscular administration of azaperone (8 mg/kg), midazolam (0.75 mg/kg) and atropine (25 μg/kg). Twenty minutes later, the animals received inhalation induction of anesthesia by sevoflurane (up to 6% end-tidal concentration) and an ear vein was cannulated (22G Abbocath, Abbott Medical, Baar/Zug, Switzerland). Animals then received fentanyl (2 μg/kg) and atracurium (0.5 mg/kg) before performing laryngoscopy and tracheal intubation with a 5.5 cuffed tube. Maintenance of anesthesia was achieved by i.v. infusion of propofol (10–15 mg⋅kg^–1^⋅h^–1^), fentanyl (10 μg⋅kg^–1^⋅h^–1^) and midazolam (0.1 mg⋅kg^–1^⋅h^–1^). After ensuring adequate levels of anesthesia and analgesia, atracurium was infused (1 mg⋅kg^–1^⋅h^–1^) to provide neuromuscular blockade. Piglets were mechanically ventilated in a supine position (Servo-I, Maquet Critical Care, Solna, Sweden) with PRVC mode (VT: 7 ml/kg, RR: 30–35/min, FiO_2_: 0.4, PEEP: 5 cmH_2_O). The femoral artery and jugular vein were cannulated for continuous hemodynamic measurements and blood withdrawal. The body temperature was measured by a rectal thermometer (Thermalert TH-8, Physitemp, Clifton, NJ, United States) and maintained at approximately 38°C using a heating pad (Miostar, Zurich, Switzerland).

### Assessment of gas exchange

Arterial and venous blood samples were collected simultaneously. The arterial partial pressure of oxygen (PaO_2_) and carbon-dioxide (PaCO_2_) and the central venous oxygen saturation (SvO_2_) were determined (VetScan i-STAT1, Abaxis, Union City, CA, United States). Oxygenation index was defined as PaO_2_/FiO_2_. The calculated capillary (CcO_2_), arterial (CaO_2_) and venous (CvO_2_) oxygen contents were used to determine the intrapulmonary shunt fraction (Qs/Qt) by applying the modified Berggren equation (15, 16):

$$\frac{Q_{s}}{Q_{t}}=\frac{C\,c\,O_{2}-C\,a\,O_{2}}{C\,c\,O_{2}-C\,v\,O_{2}}$$

### Measurement of lung volumes and lung clearance index

To determine the end-expiratory lung volume (EELV), a well-established helium (He) multiple-breath washout technique (EXHALYZER D; ECO Medics AG, Dürnten, Switzerland) procedure was used. EELV values were normalized to body weight (nEELV) to compensate for the differences of the sizes of the animals. In addition to EELV recordings, an indicator of ventilation homogeneity and a sensitive marker for small airway collapse, lung clearance index (LCI), was also assessed (17). LCI was calculated as the number of lung volume turnovers required to decrease the He concentration to 2.5% of the starting value (18).

### Assessment of lung aeration

Lung aeration was determined by electrical impedance tomography (EIT). An electrode belt containing 16 electrodes was placed around the chest at the fifth intercostal space and connected to a recorder (PulmoVista 500, Draeger, Lubeck, Germany).

Electrical impedance tomography (EIT) were generated by the injection of small electrical currents (5 mA/50 Hz). Images of 32 × 32 pixels were reconstructed from the EIT data using the manufacturer’s algorithm (19, 20). End-expiratory impedance values were assessed at three time points during the 2-min-long recordings and averaged. Global impedance data were extracted from these data sets and four regions of interest (ROI) defined as layers were also analyzed as the percentage of the global impedance values. The layers represent the ventral and dorsal parts of the dependent and non-dependent lung zones. Zone 1 depicts the ventral non-dependent zone; zone 2 depicts the dorsal non-dependent zone, zone 3 illustrates the ventral dependent zone and zone 4 represents the dorsal dependent zone.

### Hemodynamic monitoring

Heart rate and electrocardiogram (ECG) were recorded by PowerLab (PowerLab, ADinstruments, Oxfordshire, United Kingdom). Mean arterial pressure (MAP) and cardiac output (CO) were determined by pulse index continuous cardiac output (PiCCO, PiCCO Plus, Pulsion Medical Systems) (21, 22).

### Assessment of airway and respiratory tissue mechanics

Forced oscillation technique was used to determine the mechanical impedance spectra of the respiratory system (Z_rs_). To perform measurements the ETT was connected to a loudspeaker-in-box system. Avoiding derecruitment during the disconnection and reconnection to the ventilation system, the ETT was clamped at the end of expiration. The loudspeaker generated a small-amplitude pseudo-random signal with non-integer multiple frequency components between 0.5 and 21 Hz. The central airflow (V’) was measured at the distal end of the setup near the ETT, using a pneumotachograph (PNT 3700 Hans Rudolph Inc., Shawnee, KS, United States) connected to a differential pressure transducer (Honeywell model 24PCEFA6D, Charlotte, NC, United States). A second pressure transducer positioned 1–2 cm beyond the end of the ETT, was used to measure airway opening pressure (P_ao_). For the 10-s-long data collection, the clamp was released, then the ETT was clamped once again until the reconnection to the ventilatory circuit. Z_rs_ was calculated as P_ao_/V’ (23, 24).

The mechanical properties of the respiratory system were characterized by fitting a well-established model to the ensemble-averaged Z_rs_ spectra. The model contained a frequency-independent airway resistance (R_aw_) and airway inertance with a viscoelastic constant-phase tissue unit that incorporates tissue damping (G) and elastance (H) (25). The flow resistance of the apparatus, including the ETT, was measured and was subtracted from the reported R_aw_ values.

### Performing flow-controlled ventilation and registration of ventilation parameters

Flow-controlled ventilation (FCV) was performed by Evone^®^ ventilator (Ventinova Medical, Eindhoven, Netherlands). Regarding to the manufacturer’s instructions FCV can be applied through a special ultra-thin endotracheal tube (Tritube^®^) or a standard endotracheal tube (ETT) supplemented with an adapter which measures the airway pressure in the trachea, at the distal end of the setup. To standardize FOT measurements and avoid the risk of derecruitment due to reintubation, we used a standard ETT during the whole protocol. Tracheal pressure was continuously recorded also at the proximal end of the ETT by PowerLab (PowerLab, ADinstruments, Oxfordshire, United Kingdom). Peak inspiratory pressure (PIP), positive end-expiratory pressure (PEEP), flow and inspiratory to expiratory ratio can be set manually on the Evone ventilator. The setting of the respiratory rate on the ventilator is determined by different parameters such as flow, ventilatory pressures, minute volume. As the ventilator registers the mechanical parameters of the respiratory system (resistance, compliance) periodically, a 2-s-long inspiratory hold was interposed after every 10–12 breathing cycle. This mechanism resulted an elevated respiratory rate between the inspiratory holds to ensure the minute volume.

### Study protocol

**FIGURE 1:**
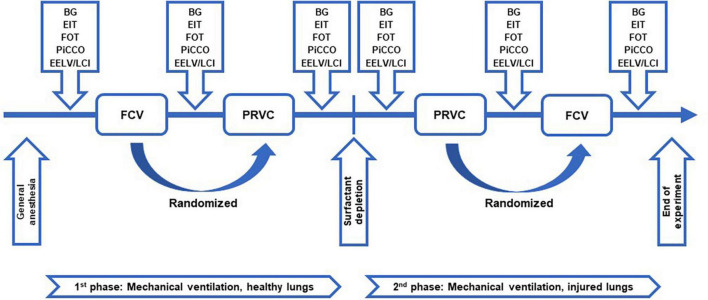
Study protocol. Schematic representation of the study protocol. FCV, flow-controlled ventilation mode; PRVC, pressure-regulated volume control ventilation mode; BG, arterial and venous blood sample collection and blood gas measurements; EIT, determination of lung aeration by electrical impedance tomography; FOT, measurement of respiratory mechanical parameters by forced oscillation technique; PiCCO, hemodynamical data collection by pulse index continuous cardiac output; EELV/LCI, measurement of end-expiratory lung volume and lung clearance index.

The study protocol is represented in Figure 1. After anesthesia induction, instrumentation and surgical preparations, animals were ventilated with PRVC (VT: 7 ml/kg, RR: 30–35/min, FiO_2_: 0.4, PEEP: 5 cmH_2_O, I:E ratio of 1:1.5) until a steady-state hemodynamic and ventilation condition had been established. A lung hyperinflation maneuver (by inflating the respiratory system to a peak pressure of 30 cmH_2_O for 8–10 s) was applied at the beginning of the protocol to standardize the volume history. A set of baseline (BL) data were then collected. Animals were then randomized to be ventilated for 1 h by either FCV (flow: 10 l/min, peak pressure: targeting a tidal volume of 7 ml/kg and normocapnia, FiO_2_: 0.4, PEEP: 5 cmH_2_O, I:E ratio of 1:1) or PRVC (VT: 7 ml/kg, RR: 30–35/min, FiO_2_: 0.4, PEEP: 5 cmH_2_O, I:E ratio of 1:1.5). As a cross-over design, the ventilation modality was then changed for another 1-h period and data collection was repeated. In the next phase of the protocol, surfactant depletion was applied by whole lung lavage. This intervention was achieved by instillation of warm 0.9% saline solution (20 ml/kg) into the ETT with subsequent withdrawal of intratracheal fluid by gentle manual suctioning. This surfactant depletion procedure was repeated 5–10 times, with the animal being reconnected and ventilated between the maneuvers. After the lavage, lung injury was further facilitated by applying a 10-min period of injurious ventilation (VT: 12 ml/kg, FiO_2_: 1, PEEP: 0 cmH_2_O) (26–28). To ensure minute volume and normocapnia, the respiratory rate was decreased for the period of injurious ventilation. The same data collection sequence with another randomization as to the order of ventilation modalities was repeated in the injured lungs as described above. To prevent potential lung derecruitment subsequent to ventilation mode switch, clamping of the ETT and a limited disconnection time of 6-8 s were provided. Lung hyperinflation maneuvers were repeated at the beginning of each ventilation stage. At the end of the protocol, animals were euthanized by a single i.v. injection of sodium pentobarbital (200 mg/kg).

### Exclusion criteria

All the experimental animals were included in the final data analysis.

### Sample size estimation

Since the FCV was provided through a normal ETT to allow the assessment of respiratory mechanics, respiratory system elastance was used to estimate sample size for two-way repeated measures ANOVA, based on similar previous experimental conditions (29). We considered 20% differences as clinically relevant between the ventilation modalities, assuming a coefficient of variation of 10. This analysis showed that at least nine animals were required to detect statistically significant changes with a statistical power of 0.9 and a two-sided alpha error of 0.05. Considering the potential drop-out rate of about 10%, we attempted to include 10 animals.

### Statistical analyses

Data are expressed as mean and standard deviation (SD). The Shapiro-Wilk test was used to test normality. Two-way repeated measures ANOVA with Holm–Šidák post-hoc analyses were applied to determine the differences between different ventilation modalities in healthy and surfactant-depleted lungs. The statistical tests were performed with SigmaPlot (Version 13, Systat Software, Inc., Chicago, IL, United States). Statistical analyses were conducted with a significance level of *p* < 0.05.

## Results

**FIGURE 2:**
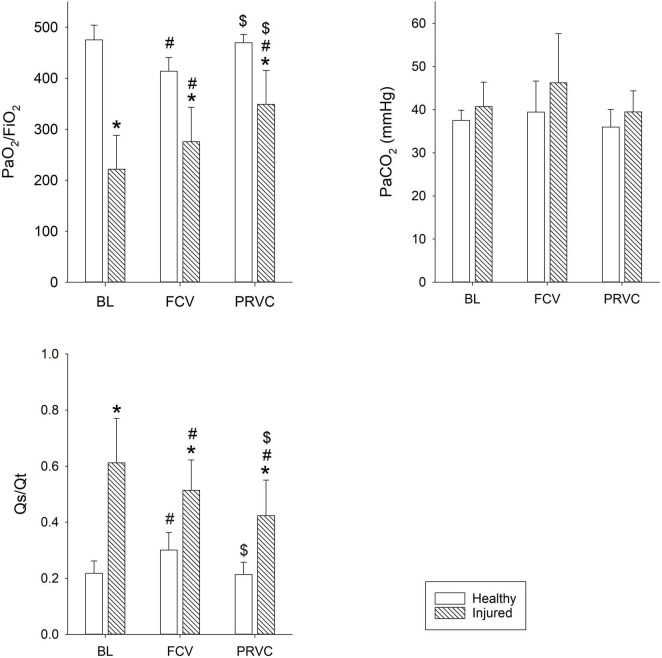
Gas exchange parameters. Gas exchange values during different ventilation stages in healthy and surfactant-depleted lungs. Empty bars represent healthy lung; patterned bars represent injured lung. *: *p* < 0.05 vs. same stage in healthy lung; #: *p* < 0.05 vs. BL within phase; $: *p* < 0.05 vs. FCV within phase. BL, baseline period; FCV, flow-controlled ventilation phase; PRVC, pressure-regulated volume control ventilation phase; PaO_2_/FiO_2_, fraction of inspired oxygen; Qs/Qt, intrapulmonary shunt fraction; PaCO_2_, partial pressure of carbon dioxide in arterial blood.

The gas exchange parameters obtained during FCV and PRVC modalities in healthy and injured lungs are summarized in Figure 2. Significantly lower PaO_2_/FiO_2_ values were observed during FCV in healthy and injured lungs when compared to prolonged PRVC (*p* = 0.009 and *p* < 0.01, respectively), which was associated with opposite changes in Qs/Qt (*p* = 0.05 and *p* = 0.007, respectively). A significant increase in PaO_2_/FiO_2_ and a decrease in Qs/Qt could be observed between healthy and surfactant-depleted lungs in each ventilation stage. The PaCO_2_ remained stable under all ventilation modes regardless of the injury.

**FIGURE 3:**
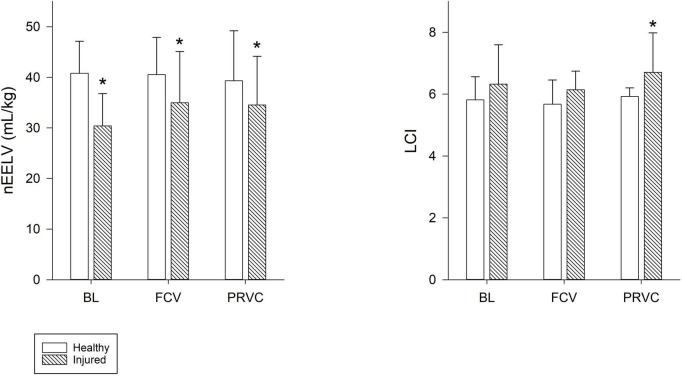
End-expiratory lung volume and lung clearance index. Normalized end-expiratory lung volume and lung clearance index values during the different ventilation modalities for healthy and injured lungs. Empty bars represent healthy lung; patterned bars represent injured lung. *: *p* < 0.05 vs. same stage in healthy lung. BL, baseline period; FCV, flow-controlled ventilation phase; PRVC, pressure-regulated volume control ventilation phase; nEELV, end-expiratory lung volume normalized to bodyweight; LCI, lung clearance index.

Figure 3 summarizes the nEELV and LCI values. While surfactant depletion led to significant decrease in nEELV (*p* < 0.001) and increase in LCI (*p* < 0.05), ventilation modality had no significant effect on these lung function variables. In injured lungs, LCI showed significantly higher values during PRVC (*p* = 0.022) and tended to be higher in FCV compared to healthy values (*p* = 0.074).

**FIGURE 4:**
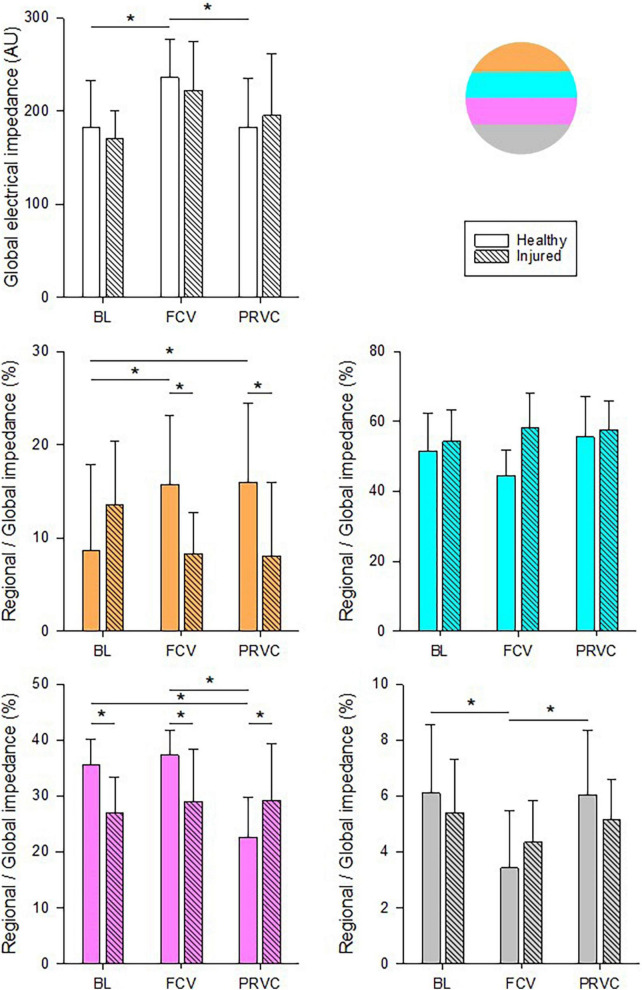
Chest electrical impedance and aeration. Global absolute impedance values and regional relative contributions during different ventilation stages in healthy and surfactant-depleted lungs. The different colors represent the lung regions in supine position. Empty and color filled bars represent healthy lung; patterned bars represent injured lung. *: *p* < 0.05. BL, baseline period; FCV, flow-controlled ventilation phase; PRVC; AU, arbitrary unit.

Impedance values for the entire lung and the relative contribution of each ROI are demonstrated in Figure 4. The global lung aeration was increased in FCV in healthy lungs compared to BL and PRVC stages (*p* < 0.05, respectively) in accordance with an increased relative regional aeration of nondependent, ventral lung zones (*p* = 0.002 and *p* < 0.001, respectively). Conversely, impedance decreased during FCV in healthy lungs in the dependent, dorsal lung regions compared to BL and PRVC (*p* = 0.005 and 0.009).

Table 1 summarizes the ventilation, respiratory mechanical and hemodynamic parameters. Increased RR and VT values could be observed during FCV compared to BL and PRVC both in healthy and injured lungs (*p* < 0.05 for all). Significant difference was evidenced in PIP, R_aw_, G and H between healthy and injured lungs at each ventilation stage (*p* < 0.05 for all). During FCV and PRVC of injured lungs, G values were significantly lower compared to BL (*p* = 0.03 and *p* = 0.007). Heart rate, MAP, CO and SvO_2_ remained stable in FCV and PRVC ventilation modes regardless of the injury.

**TABLE 1:** Ventilation, respiratory mechanical and hemodynamical parameters during different ventilation stages in healthy and surfactant-depleted lung.

	Healthy lung			Injured lung		
	BL	FCV	PRVC	BL	FCV	PRVC
RR (breaths/min)	35.0 ± 4.3	50.0 ± 7.4^#^	35.4 ± 5.0^$^	30.0 ± 4.1	51.9 ± 9.8^#^	35.0 ± 5.3^$^
VT (ml)	82.0 ± 3.7	87.6 ± 6.9^#^	83.1 ± 2.2^$^	83.3 ± 2.1	88.8 ± 6.9^#^	83.4 ± 1.8^$^
PIP (cmH_2_O)	14.5 ± 2.7	16.1 ± 1.8	14.0 ± 1.3	25.1 ± 5.1*	24.7 ± 5.9*	23.6 ± 6.1*
R_aw_ (cmH_2_O⋅s⋅l^–1^)	3.0 ± 0.8	3.1 ± 1.3	2.82 ± 0.8	4.3 ± 1.4*	4.5 ± 1.8*	4.0 ± 1.1*
G (cmH_2_O/l)	17.3 ± 3.5	17.0 ± 4.1	15.7 ± 2.9	27.2 ± 10.3*	25.9 ± 9.3*^#^	23.4 ± 6.3*^#^
H (cmH_2_O/l)	93.0 ± 14.3	91.8 ± 21.9	88.0 ± 15.6	146.7 ± 27.5*	148.2 ± 38.2*	139.9 ± 32.0*
HR (beats/min)	108 ± 17	91 ± 6	87 ± 9	101 ± 9	108 ± 20	105 ± 14
MAP (mmHg)	76 ± 4	77 ± 7	79 ± 12	76 ± 11	76 ± 8	72 ± 7
CO (l/min)	1.7 ± 0.3	1.5 ± 0.3	1.5 ± 0.3	1.6 ± 0.3	1.7 ± 0.5	1.5 ± 0.2
SvO_2_ (%)	88 ± 7.6	84 ± 8.0^#^	84 ± 6.5^#^	78 ± 7.3*	79 ± 10.2	85 ± 5.5^#^

*: p < 0.05 vs. same stage in healthy lung; #: p < 0.05 vs. BL within phase. $: p < 0.05 vs. FCV within phase. BL, baseline period; FCV, flow-controlled ventilation phase; PRVC, pressure-regulated volume control ventilation phase; RR, respiratory rate; VT, tidal volume; PIP, peak inspiratory pressure; R_aw_, airway resistance; G, tissue damping; H, tissue elastance; HR, heart rate; MAP, mean arterial pressure; CO, cardiac output; SvO_2_, central venous oxygen saturation. In case of FCV, RR was modulated by interposing an end-inspiratory pause for 1–2 s in every 10 cycles.

## Discussion

In the present study we investigated the feasibility of FCV in a pediatric experimental model. This ventilation modality ensured adequate gas exchange at a driving pressure comparable to that observed with PRVC and maintained lung aeration without compromising respiratory mechanics and hemodynamics. Compared to PRVC, FCV maintained static lung volume without enhancing ventilation heterogeneities and improved global lung aeration. While application of FCV did not affect CO_2_ elimination, a mild but significant decrease in oxygenation index and an increase in intrapulmonary shunt were observed with this modality as compared to PRVC.

Due to lower energy dissipation, FCV has been proposed as an attractive alternative to conventional protective ventilation modalities (7, 8). A further advantage of FCV is that, when it is applied through a Tritube^®^, more space is available for surgical interventions in the upper airways (12, 30). The present study was not designed to use this latter technical benefit, but focused on the comprehensive description of gas exchange, static lung volume, respiratory mechanics, and lung aeration. Therefore, FCV was applied through a standard ET tube that enabled the assessment of all these lung function variables and allowed their comparison with those obtained under a conventional protective ventilation modality.

Previous preclinical and clinical studies performed with adult patients or large mammals demonstrated the beneficial effects of FCV on gas exchange and respiratory mechanics compared to conventional mechanical ventilation modes (9, 31). The superiority of FCV over the conventional pressure-controlled ventilation was evidenced by the maintenance of oxygenation in various animal models of lung disorders, including ARDS (32), and one lung ventilation (11). Under clinical conditions, the benefit of FCV on gas exchange was also demonstrated for adults with healthy lungs (33). More recently, FCV has been advocated in a patient with COVID-19-induced lung injury (34). Previous studies and case reports revealed the applicability of FCV in children, using the Ventrain^®^, which is a hand-held ventilation device, primarily used in emergency situations (35). However, the feasibility of FCV performed by Evone^®^ ventilator in a pediatric model hasn’t been investigated yet. This modality proved to be applicable even through a normal pediatric-size endotracheal tube adapted for children of 4 to 5 years of age. Therefore, the results of the present study could be translated to a pediatric clinical setting without the application of a special airway device.

Applying FCV in healthy lungs maintained adequate CO_2_ removal, lung volume and ventilation distribution with an overall increase in lung aeration as reflected in the increase in electrical impedance (Figure 4). In contrast, a significant decrease in the oxygenation index was observed compared to PRVC: however, PaO_2_ remained in the upper range of the physiological values. This decrease can be attributed to the extra ventilation dead-space added to the circuit during FCV application. However, this extra dead-space was not evidenced in increased PaCO_2_ possibly due to the somewhat elevated minute ventilation with FCV and/or by redistribution of ventilation from the dorsal to the ventral dependent zones (Figure 4). These compensations may not have been sufficient to achieve the equilibrium between the airway opening and the alveoli, leading to a decreased diffusion time for oxygen. Since PaO_2_ is the primary determinant of Qs/Qt, the slightly decreased oxygenation was then reflected in a mild elevation in intrapulmonary shunt.

In agreement with previous results, the applied surfactant depletion and injurious ventilation mimic the main features of lung injury, including moderate decreases in oxygenation, increased intrapulmonary shunt, diminished static lung volume and elevated ventilation heterogeneity (26, 36). These functional changes were also reflected in redistribution of lung aeration from the dorsal to the ventral regions (Figure 4). In this restrictive lung model, FCV was as effective as PRVC in maintaining adequate ventilation and gas exchange. Compared to baseline, application of FCV improved oxygenation index and intrapulmonary shunt but to a lesser extent than PRVC (Figure 2). While it cannot be excluded that this observation may result partly from a time effect, the lack of difference between the two ventilation modalities confirms that FCV provides adequate ventilation for an injured lung, even in a pediatric setting.

Although the just-weaned piglet model was chosen in the present study as a pediatric model to investigate the differences between FCV and PRVC, the supine positioning of the animal is not physiological, which may have affected our findings. As a consequence of this body positioning, the intrapulmonary shunt was somewhat elevated regardless of the ventilation modality. Nevertheless, the differences between PRVC and FCV were not affected by this methodological bias and the findings of the present study allow the comparisons of the ventilation modalities on lung function and structure. A further technical consideration is that although, we aimed to compare a conventional volume control mode to flow-controlled ventilation, the setting of an exact tidal volume proved challenging due to the fact that the delivered volume in flow-controlled mode is determined by the flow-rate and the driving pressure. A further methodological consideration may be related to the limitations of the acute lung injury model, as the adverse alterations do not incorporate all the pathophysiological processes encountered in ARDS or chronic inflammatory lung diseases. Finally, since the goal of the present study was the characterization of the differences in lung function and structure, FCV was not applied through the Tritube^®^, which has advantageous and specific indications in clinical practice. Further research on FCV using the Tritube^®^, is necessary before promoting the implementation of this ventilation modality for children.

## Conclusion

In summary, the results of the present study demonstrated that the use of FCV can be extended to pediatric populations with healthy and injured lungs. Although oxygenation was inferior in FCV, it did not reach a critical level and adequate gas exchange was ensured. The lack of major differences in lung structure and function between FCV and PRVC suggests that FCV may be considered as an alternative protective ventilation modality in this setting. Further research is required to confirm the clinical value of long-term application of FCV in pediatric patients. Furthermore, the use of the Tritube^®^, has the promise of adding a novel tool to the armamentarium for the ventilation of children with difficult airways.

## Author’s note

Preliminary data for this study were presented as a poster at Euroanaesthesia 2022 congress.

## Data availability statement

The raw data supporting the conclusions of this article will be made available by the authors, without undue reservation.

## Ethics statement

This animal study was reviewed and approved by Animal Welfare Committee of the Canton of Geneva (Rue Adrien-Lachenal 8, 1207 Genève) and the Experimental Committee of the University of Geneva, Rue du Général Dufour 24, 1211 Geneva, Switzerland (no. GE 66/20; Prof. Doron Merkler, Academic Director of Animal Experimentation), on 12 May 2020.

## Author contributions

ÁB, RS, WH, and GA: conception and design of research. ÁB, JD, RS, and GA: performed the experiments. ÁS, ÁB, RS, FP, WH, and GA: analyzed the data and interpreted the results of the experiments. ÁS and FP: prepared the figures. ÁS, FP, and WH: drafted the manuscript. All authors edited, revised the manuscript, and approved the final version.

## Abbreviations

ANOVA: analysis of variance
ARRIVE: animal research reporting of *in vivo* experiments
AU: arbitrary unit
BG: arterial and venous blood sample collection and blood gas measurements
BL: baseline
CaO_2_: calculated arterial oxygen content
CcO_2_: calculated capillary oxygen content
CO: cardiac output
CvO_2_: calculated venous oxygen content
ECG: electrocardiogram
EELV: end-expiratory lung volume
EIT: electrical impedance tomography
ET tube/ETT: endotracheal tube
FCV: flow-controlled ventilation
FiO_2_: fraction of inspired oxygen
G: respiratory tissue damping
H: respiratory tissue elastance
HR: heart rate
i.v.: intravenous
I:E ratio: inspiratory-to-expiratory time ratio
LCI: lung clearance index
MAP: mean arterial pressure
nEELV: end-expiratory lung volume normalized to body weight
PaCO_2_: arterial partial pressure of carbon dioxide
P_ao_: airway opening pressure
PaO_2_: arterial partial pressure of oxygen
PaO_2_/FiO_2_: oxygenation index
PEEP: positive end-expiratory pressure
PiCCO: pulse index continuous cardiac output
PIP: peak inspiratory pressure
PRVC: pressure-regulated volume control ventilation
Qs/Qt: intrapulmonary shunt fraction
R_aw_: airway resistance
ROI: region of interest
RR: respiratory rate
SD: standard deviation
SvO_2_: central venous oxygen saturation
V’: central airflow
VT: tidal volume
Z_rs_: input impedance of the total respiratory system.
