# Erratum: Flow-controlled ventilation maintains gas exchange and lung aeration in a pediatric model of healthy and injured lungs: A randomized cross-over experimental study

**DOI:** 10.3389/fped.2023.1177367

**Published:** 2023-03-17

**Authors:** Frontiers Production Office

**Affiliations:** Frontiers Media SA, Lausanne, Switzerland

**Keywords:** flow-controlled ventilation, pediatric model, respiratory mechanics, gas exchange, lung aeration, respiratory distress syndrome

An Erratum on Flow-controlled ventilation maintains gas exchange and lung aeration in a pediatric model of healthy and injured lungs: A randomized cross-over experimental study By Schranc Á, Balogh ÁL, Diaper J, Südy R, Peták F, Habre W and Albu G. (2022) Front. Pediatr. 10:1005135. doi: 10.3389/fped.2022.1005135

An omission to the funding section of the original article was made in error. The following sentence has been added: “Open access funding was provided by the University of Geneva”.

The original version of this article has been updated.

